# Editorial: Exploring mechanisms and alleviation strategies for ammonium toxicity in plants with a focus on abiotic stress interactions

**DOI:** 10.3389/fpls.2026.1944344

**Published:** 2026-08-11

**Authors:** Ali Raza, Hafiz Hassan Javed, Weichao Wang, Muhammad Ahsan Asghar

**Affiliations:** 1 State Key Laboratory of Rice Biology, China National Rice Research Institute, Hangzhou, China; 2 South China Sea Institute of Oceanology, Chinese Academy of Sciences, Guangzhou, China; 3 The Key Laboratory of Oasis Eco-Agriculture, Xinjiang Production and Construction Crops, Shihezi University, Shihezi, Xinjiang, China; 4 Department of Food Science, Aarhus University, Aarhus, Denmark

**Keywords:** ammonium toxicity, ion toxicity, nitrate-ammonium transporter, nitrogen assimilation, nitrogen use efficiency

Ammonium (NH_4_^+^) and nitrate (NO_3_^-^) are the two primary inorganic nitrogen (N) sources that regulate plant growth, metabolism, and crop productivity (Raza et al., 2021). Although NO_3_^-^ is generally considered the preferred N source for most plant species, numerous studies have shown that an appropriate combination of NH_4_^+^ and NO_3_^-^ can enhance plant nutrient uptake, photosynthetic performance, and overall physiological and biochemical processes (Raza et al., 2022). Ammonium toxicity refers to the physiological and metabolic disorder that occurs when plants accumulate excessive amounts of NH_4_^+^ under sole or dominant nitrogen form, leading to impaired growth, reduced biomass, chlorosis, inhibited root development, and decreased crop productivity (Britto and Kronzucker, 2002). The underlying mechanisms include cytosolic/rhizosphere acidification from ammonium assimilation, disrupting carbon-nitrogen metabolism, ion homeostasis, cellular pH, and redox balance (Esteban et al., 2016; Yue et al., 2026). These disturbances trigger oxidative stress, impair photosynthesis, and alter hormonal signalling, ultimately compromising plant growth and stress resilience. Understanding these mechanisms is crucial for developing strategies to improve nitrogen use efficiency and enhance crop tolerance to ammonium-rich environments. The severity of this toxicity is strongly influenced by environmental factors such as light intensity, oxygen availability, nutrient composition, and soil pH, which regulate NH_4_^+^ uptake, assimilation, and cellular homeostasis (Urlić et al., 2017). Moreover, increasing fertilizer inputs, intensive cropping systems, and changing environmental conditions have intensified plant exposure to ammonium stress, making it an important challenge for sustainable agriculture. Previous studies have identified several mechanisms underlying ammonium toxicity, including photosystem disruption, rhizosphere acidification, excessive reactive oxygen species (ROS) production, depletion of essential inorganic cations, and interactions with other abiotic stresses such as hypoxia, light stress, and nutrient imbalance (Li et al., 2014; Xiao et al., 2023). Nevertheless, critical knowledge gaps remain in understanding the integrated physiological, molecular, and genetic networks that regulate plant responses to ammonium toxicity and in developing effective, sustainable strategies to mitigate its detrimental effects while improving nitrogen use efficiency under diverse agricultural conditions.

In this Research Topic, the articles provided the new insights into the fundamental mechanisms of ammonium toxicity and highlighted emerging approaches that enable plants to survive under ammonium-associated stress conditions. These studies collectively presented a comprehensive perspective on ion homeostasis, transporter coordination, transcriptomics regulation, genetics improvement and, environmental factors influencing ammonium toxicity in plants. Among them, Rivero-Marcos reviewed the functional interactions between nitrate (NO_3_^-^) and ammonium (NH_4_^+^) transport systems and their roles in maintaining nitrogen acquisition and cellular pH homeostasis. This study revealed the central role of the nitrate transceptor NRT1.1 not only in NO_3_^-^ uptake and signaling but also in modulating NH_4_^+^ tolerance through both nitrate-dependent and nitrate-independent pathways. It emphasizes that NH_4_^+^ uptake is closely linked to proton release, which lowers apoplastic and rhizosphere pH and disrupts nutrient homeostasis. Furthermore, regulatory components such as CIPK23 kinase are proposed as key modulators coordinating both NRT1.1 and AMT activities for nitrogen uptake according to external availability. Author also discussed the role of pH-responsive transcription factors such as STOP1, which integrate low-pH signaling with nutrient transport regulation. Together, these findings suggest that NH_4_^+^ toxicity is not solely caused by NH_4_^+^ overaccumulation but results from disrupted transporter coordination, proton imbalance, and altered signaling networks.

Improving nitrogen use efficiency through genetic approaches represents another promising avenue for sustainable crop production. Despite significant advances in understanding the physiological mechanisms of ammonium toxicity, the molecular regulators that coordinate nitrogen uptake and stress tolerance remain incompletely understood. Recent studies have identified the transporter channel complex formed by SLAH3 and NRT1.1 as a promising target for mitigating ammonium toxicity while improving nitrogen use efficiency (Wang et al., 2020). Similarly, Zhao et al. provide important mechanistic insights into how intraspecific hybridization enhances NH_4_^+^ uptake efficiency in *Saccharum spontaneum*. Their analyses revealed that hybrid progenies exhibited a specific increase in NH_4_^+^ uptake without a parallel increase in nitrate acquisition, suggesting a preferential shift toward the energetically more efficient NH_4_^+^ assimilation pathway. Transcriptomic and co-expression network analyses further showed a systemic transcriptional reprogramming characterized by the downregulation of nitrate assimilation genes and the restructuring of carbon metabolic pathways for ammonium assimilation. Notably, the study identified a multi-layered regulatory network consisting of transcription factors (AP2/EREBP, bHLH, and MYB), nitrogen metabolism enzymes such as glutamate decarboxylase (GAD), trehalose metabolism regulators (TPP/TPS), and root developmental genes. Furthermore, at the molecular level, Liu et al. investigated the response of Tartary buckwheat roots to high ammonium stress using physiological, transcriptomic, and genomic analyses. By comparing different genotypes, the authors identified genotype-dependent 19 NH_4_^+^ responsive small secreted peptide genes, particularly FtCLE7 and FtCEP3, revealing a potential signalling role in root stress regulation. By integrating genomic resequencing data, the authors detected genotype-specific variants linked to ammonium tolerance, providing valuable molecular targets for breeding.

Besides genetic regulation, environmental factors also play an important role in mitigating ammonium toxicity. Recent studies suggest that optimizing the photo environment represents a promising and sustainable approach to mitigate abiotic stresses, including ammonium toxicity (Ma et al., 2022). Furthermore, Li et al. demonstrated that blue LED light play an important role in mitigating ammonium (NH_4_^+^) toxicity in rapeseed (*Brassica napus* L.), a crop known for its sensitivity to sole NH_4_^+^ nutrition. The authors designed a hydroponic experiment with three nitrogen regimes (100% NO_3_^-^, 50% NH_4_^+^ + 50% NO_3_^-^, and 100% NH_4_^+^) under white and blue LED light conditions. Results revealed that plants grown under sole ammonium with white light exhibited severe toxicity symptoms, impaired photosynthesis, reduced biomass, and excessive accumulation of reactive oxygen species (ROS). In contrast, blue light significantly alleviated these adverse effects by improving root morphology, increasing chlorophyll content, and enhancing photosynthetic efficiency. Importantly, blue light promoted ammonium assimilation by increasing the activities of glutamine synthetase (GS), glutamate synthase (GOGAT), and glutamate dehydrogenase (GDH), thereby reducing free ammonium accumulation and its toxic effects. Their findings concluded that light quality can serve as a practical and environmentally friendly approach for improving ammonium tolerance, particularly in ammonium-sensitive species.

It has been reported that, environmental stress factors such as salinity, water logging, and drought recurrently increase ion toxicity (Yanqing et al., 2026; Zhang et al., 2025). Zhang et al. summarized the current knowledge on how NH_4_^+^ toxicity particularly severe under waterlogged conditions, where oxygen deficiency suppresses nitrification and promotes ammonium accumulation as the dominant nitrogen form in soil. Under these conditions, excessive NH_4_^+^ primarily affects roots by inhibiting primary root growth through disruption of cell division and elongation in the root apical meristem. The review summarizes that NH_4_^+^ toxicity is regulated by multiple interconnected mechanisms, including controlled ammonium uptake via AMT transporters, with CIPK23-mediated phosphorylation serving as a critical negative regulatory mechanism to prevent overaccumulation. Excess NH_4_^+^ also triggers reactive oxygen species (ROS) accumulation, particularly H_2_O_2_, which disturbs redox homeostasis and intensifies root growth inhibition. Furthermore, hormonal crosstalk involving auxin, ABA, ethylene, and nitric oxide coordinates root architectural changes and stress adaptation, while genes (VTC1, LPR2) play important roles in linking ammonium stress to root development, ROS detoxification, and ion homeostasis. In general, the review provides a mechanistic framework showing how plants integrate transporter regulation, ROS balance, and hormonal signalling to adapt to ammonium-rich and waterlogged environments.

Overall, this Research Topic collected articles demonstrated that ammonium toxicity is a multifaceted phenomenon involving transporter coordination, ion homeostasis, environmental interactions, metabolic regulation, and genetic control. The findings presented herein illustrate how plants integrate physiological and molecular mechanisms to maintain nitrogen balance under stressful conditions and provide promising strategies for improving crop resilience and nitrogen use efficiency. Future research integrating multi-omics approaches, environmental factors, and breeding technologies will further advance our understanding of ammonium toxicity and contribute to the development of climate-resilient and resource-efficient agricultural systems. We believe that this Research Topic will serve as an important reference for future studies on ammonium nutrition and its interactions with abiotic stresses, ultimately supporting sustainable crop production in changing environments.
